# Pacemaker-induced Cardiomyopathy in Patients with Coronary Artery Disease: A Report of Three Cases

**DOI:** 10.19102/icrm.2024.15011

**Published:** 2024-01-15

**Authors:** Enes Elvin Gul, Muhammad Salman Ghazni, Gehad Gamal

**Affiliations:** 1 Division of Cardiac Electrophysiology, Madinah Cardiac Centre, Madinah, Saudi Arabia

**Keywords:** Cardiac resynchronization therapy, coronary artery disease, pacemaker-induced cardiomyopathy

## Abstract

Pacing-induced cardiomyopathy (PICM) is defined as a drop in left ventricular ejection fraction (LVEF) in the setting of chronic, high-burden right ventricular pacing. Cardiac resynchronization therapy (CRT) and conduction system pacing (CSP) have been proposed to manage PICM. Although acute myocardial infarction has been described as a predictor of PICM, there are no guideline recommendations for CRT or CSP in patients with coronary artery disease (CAD) and preserved LVEF. In this report, we present and discuss three cases of PICM in patients with CAD and preserved LVEF.

## Introduction

Pacing-induced cardiomyopathy (PICM) is a potential right ventricular (RV) pacing complication. Chronic conventional RV pacing can lead to ventricular dyssynchrony and subsequent left ventricular (LV) dysfunction and heart failure (HF).^1,2^

The management of PICM remains unclear. Although there is no clear indication mentioned in the guidelines, patients with PICM can be managed by alternative pacing strategies such as device upgrade to biventricular cardiac resynchronization therapy (CRT) or other physiological pacing strategies like His-bundle pacing (HBP) and left-bundle branch area pacing (LBBAP).^3–5^

However, data on preventing PICM in patients with coronary artery disease (CAD) and preserved LV function remain scarce. Therefore, we present three cases with early-onset PICM following pacemaker implantation in patients with CAD and preserved LV function **(Table 1)**. Written informed consent was obtained from the participants of this study.

## Case presentations

### Case #1

**Figure 1:**
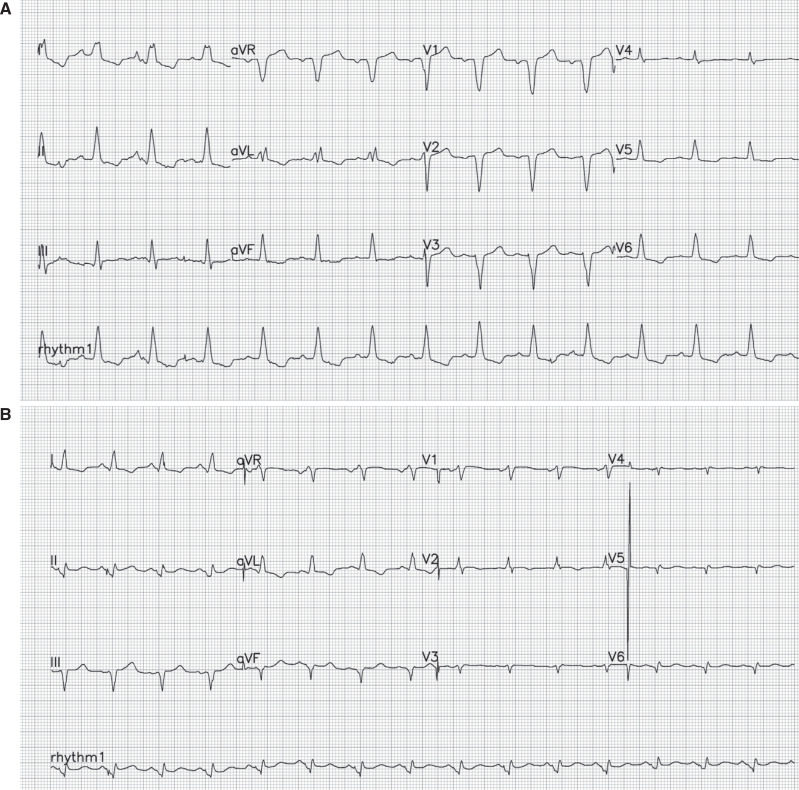
**A:** Twelve-lead electrocardiogram showing atrial sensed and ventricular pacing @ 85 bpm. The paced QRS duration is 148 ms. **B:** Twelve-lead electrocardiogram recorded after cardiac resynchronization therapy upgrade with a paced QRS duration of 117 ms.

A 71-year-old woman with a history of hypertension, diabetes, and CAD with previous left anterior descending artery (LAD) and left circumflex artery (LCx) percutaneous coronary intervention presented with syncope and documented complete heart block (CHB). She was scheduled for permanent pacemaker implantation due to alternating bundle branch block. Transthoracic echocardiography (TTE) revealed preserved LV function (left ventricular ejection fraction [LVEF], 55%). Repeat coronary angiography was performed again to rule out ischemia and showed patent stents in both the LAD and LCx, with no new critical lesions. Owing to her preserved LVEF at 55%, a dual-chamber pacemaker was chosen for implantation (May 2018). She was followed up with at the device clinic every 6 months thereafter. Paroxysmal atrial fibrillation (AF) episodes were documented, for which she took 5 mg of apixaban orally twice daily. Three years later (September 2021), the patient was admitted to our hospital with progressive shortness of breath. TTE showed a reduction in LVEF to 25%. Coronary angiography revealed no new lesions. The patient underwent a CRT upgrade due to PICM. Both paced dual-chamber and CRT electrocardiograms are shown in **Figure 1**. She was referred to the HF clinic and was placed on sacubitril/valsartan along with traditional HF drugs.

### Case #2

A 68-year-old woman with a history of hypertension and diabetes presented with chest pain, dizziness, and documented CHB with an escape rhythm of 30 bpm. A temporary pacemaker was inserted. TTE revealed a normal LVEF and an enlarged left atrial diameter. Coronary angiography revealed a mildly diseased LAD and a totally occluded right coronary artery (RCA). A dual-chamber pacemaker with RV lead placement in the septum was implanted in August 2018. She began experiencing exertional dyspnea by the end of 2020, and TTE confirmed LVEF at 15%. She was referred to the HF clinic, and optimal HF management was started. However, her symptoms did not resolve, and she was referred to an interventional cardiologist to rule out new ischemia. Coronary angiography revealed lesions similar to those in 2018. The patient was diagnosed with PICM, and a successful CRT pacemaker upgrade was performed.

### Case #3

A 68-year-old man with a history of diabetes and hypertension was admitted with CHB in December 2018. TTE revealed a normal LV function. Coronary angiography showed mild LAD disease, a normal RCA, and total occlusion of the LCx, manifesting as chronic total occlusion. A single-chamber pacemaker was implanted at that time. Four months later, the patient was admitted with pleuritic chest pain, and both RV impedance and thresholds were high. TTE showed mild pericardial effusion. Chest radiography and computed tomography confirmed an RV lead perforation. A new dual-chamber pacemaker with RV lead placement in the septum was implanted, and the perforated RV lead was removed.

The patient received device follow-up every 6 months. He only experienced non-sustained ventricular tachycardia (NSVT) episodes, and the lead parameters were within the normal limits.

In December 2022, he was admitted with decompensated HF. TTE confirmed LVEF at <30%. Coronary angiography showed moderate distal LAD and similar lesions compared to those observed in December 2018. The patient underwent a CRT defibrillator upgrade due to his history of CAD and NSVT episodes.

## Discussion

Due to a lack of definition, the prevalence of PICM ranges from 6%–25%.^6^ A recent meta-analysis reported a 12% prevalence of PICM.^7^ The definition of PICM is variable, and the most acceptable result is a reduction in LVEF to 40%–50% or a decrease in LVEF by 10%–15% after device implantation.^8,9^

Based on previous studies and meta-analyses, the main predictors of PICM were male sex, a history of myocardial infarction, chronic kidney disease, AF, baseline LVEF, native QRS duration, RV pacing percentage, and paced QRS duration.^7,9–11^

Current guidelines recommend CRT rather than RV pacing for patients with HF with reduced ejection fraction (<40%), regardless of the New York Heart Association (NYHA) class, who have an indication for ventricular pacing and high-degree atrioventricular block.^12^ All our three cases had LVEF > 40%; therefore, the dual-chamber pacemaker was preferred over CRT.

Conduction system pacing (CSP) is a new field of pacing developed to minimize or prevent dyssynchrony due to RV pacing. Both HBP and LBBAP are summarized under CSP. A clear benefit of CSP over RV pacing has been demonstrated.^13^ Studies have evaluated CRT as a treatment for PICM and reported a significant increase in LVEF after biventricular CRT.^3,8,14–16^ Three studies evaluated HBP as a treatment for PICM and showed an improvement in NYHA class and a reduction in HF hospitalizations.^4,13,17^

None of our patients underwent CSP. Although there is still no strong guideline recommendation to implant a CSP system as a first-line approach, we strongly believe that CSP system implantation would have prevented PICM. Further studies are needed to assess the benefits of CSP in patients with CAD and CHB undergoing pacemaker implantation.

CAD patients with preserved LV function may develop PICM earlier due to pre-existing disease in the native intrinsic conduction system, which may lead to prolonged QRS duration. A previous meta-analysis reported that a history of myocardial infarction was a predictor of PICM (odds ratio, 1.81).^7^

The present study has a few limitations. Unfortunately, we did not have access to follow-up echoes to determine whether LVEF improved after the CRT upgrade in any of these cases. Second, cardiac magnetic resonance imaging was not performed to determine whether there was an additional substrate that could cause a drop in LV function. Finally, it might be reasonable to monitor LVEF recovery in the first 6–12 months of new HF medications before considering CRT.

In conclusion, we aim to discuss and raise awareness regarding PICM in patients with CAD and preserved LVEF in this report.

## Figures and Tables

**Table 1:** Patient Characteristics

Patients	Age (Years)	Sex	Comorbidities	Indication	LVEF^a^	LVEF^b^	pQRSd, ms	Time to Diagnosis, Months	Intervention
1	71	Female	CAD	CHB	55	25	143	28	CRT-P upgrade
2	64	Female	CAD, DM, HTN, pAF	CHB	50	15	178	28	CRT-P upgrade
3	65	Male	CAD, DM, HTN	CHB	60	30	168	48	CRT-D upgrade

*Abbreviations:* CAD, coronary artery disease; CHB, complete heart block; CRT, cardiac resynchronization therapy; CRT-D, cardiac resynchronization therapy defibrillator; CRT-P, cardiac resynchronization therapy pacemaker; DM, diabetes mellitus; HTN, hypertension; LVEF, left ventricular ejection fraction; pAF, paroxysmal atrial fibrillation; pQRSd, paced QRS duration. ^a^LVEF at the time of implantation. ^b^LVEF at follow-up.
